# Radiation Tolerance of *Pseudanabaena catenata*, a Cyanobacterium Relevant to the First Generation Magnox Storage Pond

**DOI:** 10.3389/fmicb.2020.00515

**Published:** 2020-04-07

**Authors:** Lynn Foster, Howbeer Muhamadali, Christopher Boothman, David Sigee, Jon K. Pittman, Royston Goodacre, Katherine Morris, Jonathan R. Lloyd

**Affiliations:** ^1^Research Centre for Radwaste Disposal and Williamson Research Centre for Molecular Environmental Science, School of Earth and Environmental Sciences, University of Manchester, Manchester, United Kingdom; ^2^Department of Biochemistry, Institute of Integrative Biology, University of Liverpool, Biosciences Building, Liverpool, United Kingdom

**Keywords:** cyanobacteria, FT-IR spectroscopy, metabolic fingerprint, radiation, polysaccharide, spent nuclear fuel pond

## Abstract

Recently a species of *Pseudanabaena* was identified as the dominant photosynthetic organism during a bloom event in a high pH (pH ∼11.4), radioactive spent nuclear fuel pond (SNFP) at the Sellafield Ltd., United Kingdom facility. The metabolic response of a laboratory culture containing the cyanobacterium *Pseudanabaena catenata*, a relative of the major photosynthetic microorganism found in the SNFP, to X-ray irradiation was studied to identify potential survival strategies used to support colonization of radioactive environments. Growth was monitored and the metabolic fingerprints of the cultures, during irradiation and throughout the post-irradiation recovery period, were determined using Fourier transform infrared (FT-IR) spectroscopy. A dose of 95 Gy delivered over 5 days did not significantly affect growth of *P. catenata*, as determined by turbidity measurements and cell counts. Multivariate statistical analysis of the FT-IR spectral data revealed metabolic variation during the post-irradiation recovery period, with increased polysaccharide and decreased amide spectral intensities. Increases in polysaccharides were confirmed by complementary analytical methods including total carbohydrate assays and calcofluor white staining. This observed increased production of polysaccharides is of significance, since this could have an impact on the fate of the radionuclide inventory in the pond via biosorption of cationic radionuclides, and may also impact on downstream processes through biofilm formation and biofouling.

## Introduction

Microorganisms are ubiquitous and inhabit a wide range of environments including those with extremes of pH, temperature, pressure, availability of water, salinity, and radiation (Billi and Potts, 2002; Blanco-Rivero et al., 2005; Katz et al., 2007; Pikuta et al., 2007). The presence of microorganisms in extreme radioactive environments has been reported, for example in Spent Nuclear Fuel Ponds (SNFPs) (Diósi et al., 2003; Chicote et al., 2004, 2005; Sarró et al., 2003, 2005, 2007; Rivasseau et al., 2010, 2016; Dekker et al., 2014; Karley et al., 2017; MeGraw et al., 2018) and in contaminated land surrounding the Chernobyl nuclear reactor (Dadachova et al., 2008). Many microorganisms that are capable of withstanding high doses of radiation are also known to be able to withstand extreme environmental conditions such as desiccation, for example *Deinococcus radiodurans* and the cyanobacterium *Chroocococcidiopsis* spp. are able to survive doses of 10 kGy of ionizing radiation and periods of desiccation (Billi et al., 2000; Billi and Potts, 2002; Jolivet et al., 2003).

Studies investigating the presence of microorganisms in SNFPs across several sites, including in Spain, United States, France, India and the United Kingdom, have shown that each site exhibits a unique microbial community profile (Diósi et al., 2003; Chicote et al., 2004, 2005; Sarró et al., 2003, 2005, 2007; Rivasseau et al., 2010, 2016; Dekker et al., 2014; Karley et al., 2017; MeGraw et al., 2018). The majority of sites are dominated by *Proteobacteria*, although photosynthetic microorganisms, including eukaryotic microalgae have also been identified as dominant at some sites (Rivasseau et al., 2010). The presence of microorganisms in SNFPs is challenging since high levels of biomass can result in reduced visibility (increased turbidity) within the water column (hampering pond management), may lead to microbiologically induced corrosion (MIC) and also lead to the formation of organic-rich radioactive wastes. Bruhn et al. (2009) highlighted the potential for the formation of biofilms and MIC in the presence of elevated levels of radiation, which signals the possibility of MIC occurring on waste storage containers and fuel cladding. Indeed, several studies investigating biofilm forming organisms in ponds at the Confrentes site (Valencia, Spain) have shown the occurrence of MIC on steel coupons (Sarró et al., 2003, 2005, 2007; Chicote et al., 2004, 2005). The potential for MIC on waste storage containers and fuel cladding could have implications for the longevity of spent fuel storage in pond environments. Microorganisms have also been shown to accumulate fission products as shown with the free-living eukaryotic microalga *Coccomyxa actinabiotis*, isolated from a SNFP in research reactor in France, which has been shown to accumulate large quantities of ^137^Cs (Rivasseau et al., 2013).

The early Magnox gas cooled reactors form a significant part of the UK’s legacy nuclear fleet. The fuel rods used in these reactors were clad in a magnesium non-oxide (Magnox) alloy (Jensen and Nønbel, 1999; Gregson et al., 2011a, b; Jackson et al., 2014). Spent fuel rods containing this cladding have been stored in open air legacy storage ponds, including the First Generation Magnox Storage Pond (FGMSP) situated on the Sellafield site (Cumbria, United Kingdom), since the late 1950s (Jackson et al., 2014). Within the FGMSP, fuel storage times have been longer than anticipated and the spent fuel has been subject to extensive corrosion due to the Magnox cladding and the uranium metallic fuel having limited chemical stability in water. This extended storage has led to the formation of a high hazard radioactive environment with corroded spent nuclear fuel, pond effluent (NDA, 2016; Foster et al., 2020) and sludge (NDA, 2016). In order to provide thermal cooling and minimize further corrosion of the fuel rods and the growth of microorganisms, the pond is continuously purged with alkaline dosed demineralized water (pH ∼11.4) (Gregson et al., 2011a, b; Jackson et al., 2014). However, there is clear visible evidence for the presence of microorganisms in the pond, including events reported as “algal blooms”, which are most prominent when the purge cycling is not active (Gregson et al., 2011a, b; Konovalovaite, 2019; Foster et al., 2020).

The microbial community of this open-air legacy SNFP has recently been investigated over a three year period, including during a microbial bloom period in August 2016 (Foster et al., 2020). Over the course of the investigation, highly pigmented organisms with photosynthetic or hydrogen-metabolizing capabilities were identified. Background water samples indicated that *Proteobacteria* were the dominant microorganisms in the pond, whilst a single cyanobacterial species, *Pseudanabaena catenata*, was dominant during the bloom event (Foster et al., 2020). *Pseudanabaena* spp. are filamentous cyanobacteria, displaying a simple morphology, and lack the ability to form branches or differentiate (Acinas et al., 2009; Zhu et al., 2015). Reports of *Pseudanabaena* spp. in the scientific literature are limited, with little known about their physiology or molecular and metabolic characteristics, and although they are associated with bloom events in a range of environments, they are often overlooked (Acinas et al., 2009; Webster-Brown et al., 2015; Zhu et al., 2015; Bukowska et al., 2017; Khan et al., 2018).

The occurrence of cyanobacterial blooms in the FGMSP disrupts waste retrieval operations and downstream processes. Although the microbial community structure of the legacy SNFP has been determined, there have been no studies so far to characterize how the microorganisms are able to tolerate and colonize this highly radioactive, alkaline environment. The purpose of this study is to determine the adaptive response of a *P. catenata* culture to ionizing radiation, to help understand the potential impacts of microbial colonization on pond biogeochemistry and ultimately to inform control strategies employed onsite. It was not possible to isolate the *Pseudanabaena* species that resides in the FGMSP due to radiological safety limitations, and therefore a close-relative of the major photosynthetic microorganism was acquired from a culture collection to use in the following study. To determine the physiological and metabolic response of a *P. catenata*-containing culture to irradiation, Fourier transform infrared (FT-IR) spectroscopy and classical microbiological techniques were utilized. FT-IR spectroscopy is a metabolic fingerprinting technique that can be used to determine the phenotype in a given microbial sample, while shifts in these fingerprints can be correlated with metabolic consequences when the environment of the microbe is changed (Muhamadali et al., 2015a). Here, we demonstrate that a culture containing *P. catenata* as the sole cyanobacterial species is capable of surviving significant doses of X-ray irradiation over a period of 5 days. When grown photoautotrophically, the culture did not display any physiological differences to untreated cultures during the irradiation treatment, however, increases in polysaccharides and a reduction in chlorophyll-a (Chl-a) became more pronounced during the post-irradiation period. This study provides insights into the radiation resistance mechanisms employed by photosynthetic microorganisms related to those colonizing the FGMSP. Understanding the behavior of the microorganisms in response to radiation (and other stress responses) will underpin radiation adaptation mechanisms in extremophiles, and inform more effective control strategies to minimize microbial growth and bloom formation.

## Materials and Methods

### Culturing and X-Ray Irradiation of *P. catenata*

It was not possible to culture organisms directly from water taken from the SNFP due to radiological safety limitations. A culture of the closest known relative to the *Pseudanabaena* species detected in the pond, *P. catenata* was obtained from the NIVA Culture Collection of Algae (NIVA-CYA 152), Norway. The *P. catenata* was inoculated in unbuffered BG11 media (Culture Collection of Algae and Protozoa) and incubated at 25 ± 1°C, and shaken at 100 rpm in a light incubator with a photon flux density of 150 μmol m^–2^ s^–1^, and a 16:8 h light–dark cycle (supplied by cool fluorescent daylight lamps). Biological triplicates were prepared by inoculating 20 mL BG11 medium with *P. catenata* to a starting optical density 0.2 (OD_600__nm_). The cultures were exposed to daily doses of ionizing radiation using a Faxitron CP-160 Cabinet X-radiator (160 kV; 6 mA; tungsten target). The FGMSP has a reported dose of 5.65 Gy h^–1^ associated with the sludge, and 0.15 mGy h^–1^ associated with the pond water (Jackson et al., 2014). A dose of 1 Gy min^–1^ for 19 min per day was administered to the cultures over five consecutive days to give a total dose of 95 Gy. A further triplicate set of “no dose” controls were placed inside the irradiator, shielded by an appropriate thickness of lead, to mimic the environmental conditions within the Faxitron cabinet (e.g., any heating due to radiation). All cultures were incubated following the treatment as previously described.

### DNA Extraction and 16S rRNA Gene Sequencing of the *P. catenata* Culture

It was not possible to source an axenic culture of *P. catenata* from any culture collection for this study, therefore a non-axenic *P. catenata* culture was used for this work. The culture was characterized using 16S rRNA gene sequencing to monitor the relative abundance of all the prokaryotic microorganisms, and quantify any differences in the cultures at the end of the experiment. Samples (1 mL) of irradiated and control cultures at day 16 were passed through a sterile 0.2 μm filter using a vacuum filtration technique. DNA was then extracted using the MoBio PowerWater DNA isolation kit (MoBio Laboratories, Inc., Carlsbad, CA, United States). The 16S rRNA gene was sequenced from PCR amplicons on the Illumina MiSeq platform (Illumina, San Diego, CA, United States) targeting the V4 hyper variable region (forward primer, 515F, 5′-GTGYCAGCMGCCGCGGTAA-3′; reverse primer, 806R, 5′-GGACTACHVGGGTWTCTAAT-3′) for 2 × 250-bp paired-end sequencing (Illumina) (Caporaso et al., 2011, 2012). The Roche FastStart High Fidelity PCR System (Roche Diagnostics Ltd., Burgess Hill, United Kingdom) was used to perform the PCR amplifications (50 μL reactions) under the following conditions; initial denaturation at 95°C for 2 min, followed by 36 cycles of 95°C for 30 s, 55°C for 30 s, 72°C for 1 min, and a final extension step of 5 min at 72°C. The SequalPrep Normalization Kit (Fisher Scientific, Loughborough, United Kingdom) was used to purify and normalize the PCR products to ∼20 ng each. The PCR amplicons from all samples were pooled in equimolar ratios. The run was performed using a 4 pM sample library spiked with 4 pM PhiX to a final concentration of 10% following the method of Schloss and Kozich (Kozich et al., 2013).

A sequencing pipeline was used to divide the raw sequences into samples by barcodes (up to one mismatch was permitted). Cutadapt (Martin, 2011), FastQC^1^, and Sickle (Joshi and Fass, 2011) were used to perform quality control and trimming, whilst SPADes (Nurk et al., 2013) was used to carry out MiSeq error corrections. Forward and reverse reads were incorporated into full-length sequences with Pandaseq (Masella et al., 2012). ChimeraSlayer (Haas et al., 2011) was utilized to remove chimeras, and OTUs were generated UPARSE (Edgar, 2013) generated OTUs, that were classified by Usearch (Edgar, 2010) at the 97% similarity level, and singletons were removed. Rarefaction analysis was conducted using the original detected OTUs in Qiime (Caporaso et al., 2010). The RDP classifier, version 2.2 (Wang et al., 2007) was used to perform the taxonomic assignment.

### Growth, Chlorophyll-a (Chl-a) Concentration and pH Measurements

To quantify the total biomass in cultures by turbidity, absorbance values at 600 nm (OD_600__nm_) were recorded for 1 mL aliquots of the *P. catenata* cultures using a Jenway 6700 UV/Vis spectrophotometer (Bibby Scientific Limited, Staffordshire).

The concentration of Chl-a was determined as follows: 1 mL samples were centrifuged at 14,000 × *g* for 10 min to pellet the cells. The supernatant was then discarded and the cells re-suspended in 1 mL of 70% ethanol and incubated at room temperature for 2 h. The samples were then centrifuged at 14,000 × *g* for 10 min, the supernatant was then removed and analyzed using the Jenway 6700 UV/Vis spectrophotometer (Bibby Scientific Limited, Staffordshire). The absorbance was measured at 665 nm (Chl-a) and at 750 nm to correct for turbidity (Bellinger and Sigee, 2010). The concentration of Chl-a was then calculated using the formula of Jespersen and Christoffersen (Jespersen and Christoffersen, 1987).

The pH of the cultures was measured using a FiveEasyPlus pH meter (Mettler Toledo Ltd., Leicestershire, United Kingdom).

### Light Microscopy

All light microscopy was carried out using a Zeiss Axio Imager A1 (Carl Zeiss Microimaging 234 GmbH, Germany) light microscope fitted with an Axiocam 506 mono camera using Zen2 imaging software.

#### Cell Counts of *P. catenata*

Direct counts of *P. catenata* were carried out routinely throughout the experiment using a Sedgewick Rafter counting chamber. Ten images were taken of random sites across the samples. ImageJ was used to determine the length of filaments and individual cells. An average cell count was determined by dividing the total filament length by the average cell length. Samples were diluted with sterile BG11 medium to an appropriate concentration as required for analysis.

#### Calcofluor White Staining of β-Polysaccharides

Cells were washed twice and re-suspended in sterile normal saline (9 g L^–1^ NaCl), 5 μL of each sample was placed on a glass slide and 5 μL of calcofluor white stain (Sigma-Aldrich, Dorest, United Kingdom) was added and a cover slide placed over the sample. The samples were left to incubate for 10 min in the dark prior to being analyzed.

The auto-fluorescence of the culture was observed using filter set 00 (530–585 nm excitation and 615–4095 nm emission). Calcofluor white stain fluorescence was observed using filter set 49 (335–383 nm excitation and 420–470 nm emission).

### Carbohydrate Quantification

A total carbohydrate assay kit (Sigma-Aldrich, Dorset, United Kingdom) was used to determine carbohydrate concentrations. Prior to using the kit, the cells were prepared by washing twice with sterile normal saline solution (9 g L^–1^ NaCl), the cell pellets were flash frozen in liquid nitrogen and stored at -80°C until they were analyzed. All samples were normalized to an optical density of OD_600_ 15 (as per FT-IR preparation). Following this, a 200 μL aliquot was then centrifuged and re-suspended in the assay buffer, incubated for 10 min at room temperature. The samples were centrifuged at 14,000 × *g* for 5 min and 15 μL aliquots from the samples were used for the assay reaction and made up to 30 μL with Roche PCR grade water. The sample preparation was then carried out as detailed in the kit technical bulletin.

### Metabolic Profile of the Cultures by FT-IR Spectroscopy

Normalized samples were spotted as 20 μL aliquots onto a Bruker 96-well FT-IR silicon plate (Bruker Ltd., Coventry, United Kingdom) in triplicates, and heated to dryness (20–30 min) in an oven at 55°C (Muhamadali et al., 2015b). All FT-IR spectra were recorded in the mid-infrared range (4000–600 cm^–1^) with 4 cm^–1^ resolution and 64 spectral co-adds in absorbance mode using a HXT^TM^ module on a Bruker Equinox 55 infrared spectrometer (Muhamadali et al., 2015c).

### Multivariate Statistical Analysis

The collected FT-IR spectra were analyzed using MATLAB version 9 (The MathWorks Inc., Natick, MA). All spectra were scaled using the extended multiplicative signal correction (EMSC) method (Martens et al., 2003), followed by replacement of the CO_2_ bands (2400 to 2275 cm^–1^) with a linear trend. The pre-processed FT-IR spectral data were analyzed by the unsupervised method of principal component analysis (PCA) to reduce the dimensionality of the data and PC scores plots generated to determine any between group variations, and PC loadings plots visualized to determine which molecular vibrations were important (Wold et al., 1986).

## Results

### The Effect of X-Ray Irradiation on the Growth and Chlorophyll Concentration of the *P. catenata* Culture

**FIGURE 1:**
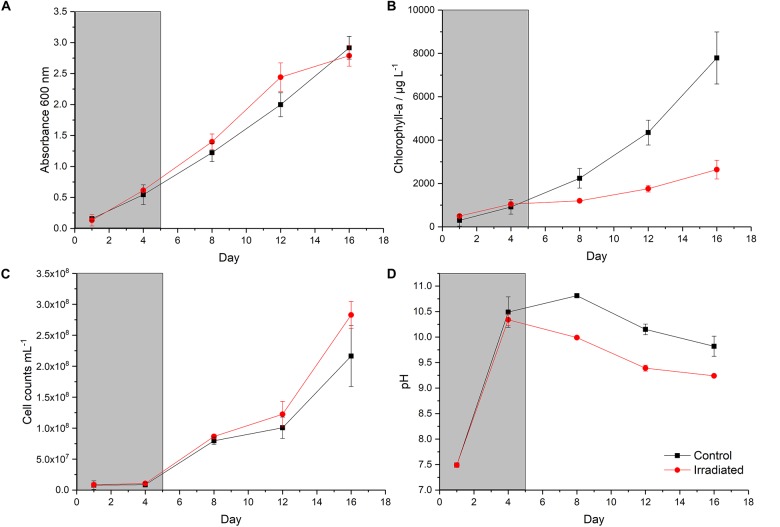
Growth of the *P. catenata* culture with and without radiation treatment: **(A)** absorbance at 600 nm; **(B)** chlorophyll-a concentration (μg L^– 1^); **(C)** mean cell counts of *P. catenata*; **(D)** pH. The gray panel indicated the period in which the irradiation treatment was being administered. Red lines are irradiated samples; black lines are control samples. Error bars are the standard deviation of three replicates.

In order to determine the effect of ionizing radiation on the growth and photosynthetic pigment characteristics of the *P. catenata* culture, the optical density at 600 nm (OD_600__nm_), cell counts and Chl-a concentration were monitored during and after the cultures were subjected to a total of 95 Gy (1 Gy min^–1^) of X-ray irradiation over a five day period (Figure 1). The optical density of *P. catenata* cultures was measured at 600 nm over a period of 16 days (Figure 1A). There was a steady increase in the optical density recorded over time for both the irradiated cultures and the unirradiated controls. The control cultures started at an average OD_600__nm_ of 0.16 and reached an OD_600__nm_ of 2.92 at day 16 whilst the irradiated culture started at a slightly lower average OD_600__nm_ of 0.13 and reached 2.79 by day 16. The overall growth profile, between irradiated and unirradiated samples, showed very similar trends indicating that the amount of biomass developed in the cultures was not significantly different. Optical density measurements were also taken at 680 nm and 750 nm, and showed the same trends as the measurements taken at 600 nm (data not shown). Since the cultures contained other prokaryotic microorganisms in addition to *P. catenata*, direct cell counts (Figure 1C) were carried out using a light microscope to ensure the trends seen in the turbidity measurements reflected the proliferation of the cyanobacterium, with its characteristic chain morphology. Observation of both irradiated and control cultures showed that *P. catenata* dominated the field of view, supporting the sequencing data which shows it was the most abundant organism in the culture. Additionally, the 16S rRNA gene sequencing showed the close relationship of the cultured *Pseudanabaena* species to one identified in the pond (Supplementary Figure 1) and that there were no changes in the community following the irradiation treatment (Supplementary Figure 2). Both the irradiated and control cultures started with around 8 × 10^6^ cells mL^–1^ and showed an increase in cell numbers over time. By day 16 the average cell counts for the irradiated cultures were 31% higher than those for the control at 2.8 × 10^8^ cells mL^–1^ and 2.2 × 10^8^ cells mL^–1^, respectively. Both the turbidity measurements and the cell counts show the same overall trends in growth, with no significant difference between the two sets of cultures observed. The concentration of Chl-a differed between the irradiated and unirradiated samples (Figure 1B). The initial Chl-a concentrations were 0.4 mg L^–1^ and 0.5 mg L^–1^ for the control and irradiated cultures, respectively. Both cultures showed similar increases in the Chl-a concentration at day 4 and whilst the irradiation treatment was still being administered. The control cultures then showed a continued increase in the Chl-a concentrations, with 7.8 μg L^–1^ measured on day 16. By contrast, the Chl-a levels in the irradiated cultures were consistently lower after day 4, at day 16 a concentration of 2.6 mg L^–1^ was recorded, which is ∼66% less than the control value. Normalization of the Chl-a concentration to cell number showed that by day 4, the concentration of Chl-a per cell increased in both treatments and reached the maximum of 1.1 × 10^–4^ μg L^–1^ cell^–1^ and 1.0 × 10^–4^ μg L^–1^ cell^–1^ for the control and irradiated cultures, respectively (Supplementary Figure 3). Both sets of cultures showed a decline in the average Chl-a concentration per cell from day 8, which plateaued toward the end of the experiment, and the irradiated culture consistently showed significantly reduced concentrations of Chl-a per cell compared to the control. At day 16 the values were 9.4 × 10^–6^ μg L^–1^ cell^–1^ and 3.7 × 10^–5^ μg L^–1^ cell^–1^, for the irradiated and control cultures, respectively. The pH of the unbuffered cultures was monitored over the course of the sampling period, both cultures started off at a pH of 7.3 which increased to pH > 10 by day 4. The pH of the irradiated culture started to decline after day 4 reaching pH 9.2 at day 16 (Figure 1D). The pH of the control sample increased to 10.8 at day 8 and then gradually fell to 9.8 at day 16. The pH of the irradiated culture was significantly lower than seen in the control culture.

### Metabolic Response of the *P. catenata* Culture to X-Ray Irradiation Determined by FT-IR Spectroscopy

**FIGURE 2:**
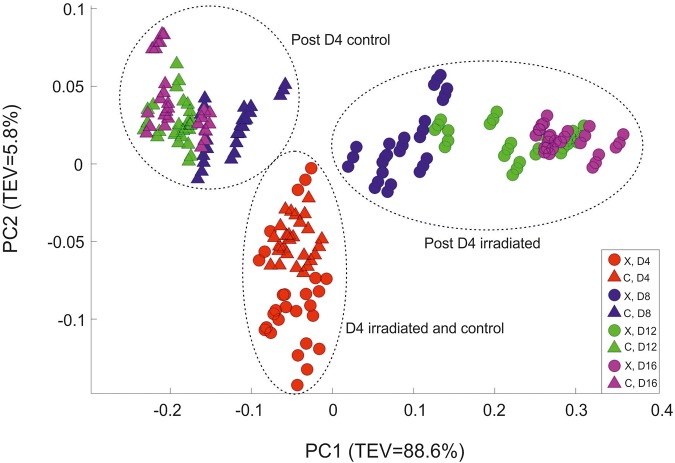
Principal component analysis scores plot of all the FT-IR spectroscopy data. Circles represent the irradiated (X) samples; triangles are control (C) samples. Color code: Red- day 4; Blue- day 8; Green- day 12; and Purple day 16. TEV = Total Explained Variance.

FT-IR spectroscopy was utilized to obtain a metabolic fingerprint of the cultures, and to explore any physiological changes associated with irradiation. A principal component analysis (PCA) scores plot of the data (Figure 2) displayed clear separation of the two treatments according to PC1, which accounted for 88.6% of the total explained variance (TEV). At day 4 both sets of samples clustered together, indicating that although one set of cells was receiving the X-ray irradiation treatment there was no significant difference between the cultures; in contrast, by day 8 there was clear separation of the samples according to the PC1 axis. The samples collected on day 4 were separated from all other samples according to the PC2 axis (TEV = 5.8%), which emphases the fact that after day 4, significant metabolic changes were occurring. The control samples from day 8 to 16 form a tight cluster (in top left part of the PCA scores plot) which was distinct from the cluster at day 4. Interestingly the irradiated samples showed continued separation over time according to PC1 (from left to right) with each time point clustering closely.

**FIGURE 3:**
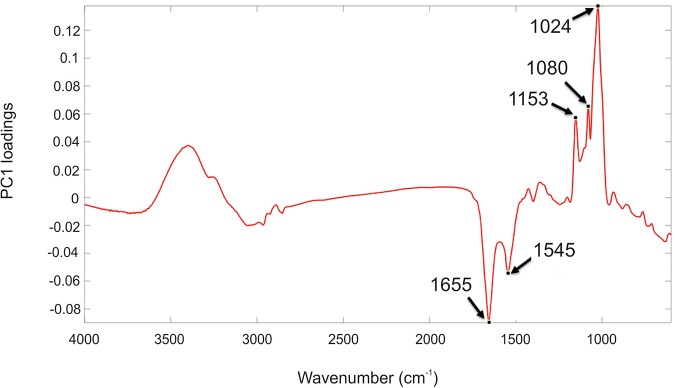
PC1 loading plot including the wavenumbers contributing to the shifts seen across PC1. Spectral features highlighted refer to the following: 1655 cm^– 1^ (amide I); 1545 cm^– 1^ (amide II); 900–1200 cm^– 1^ (carbohydrates).

**FIGURE 4:**
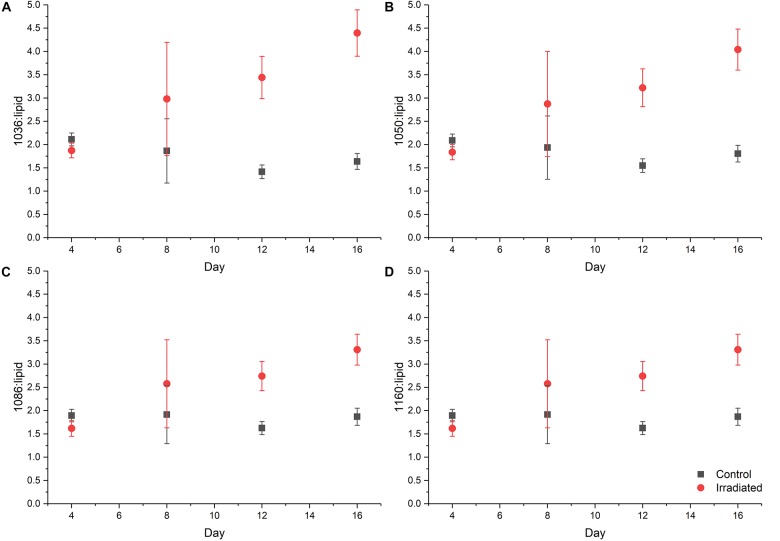
Ratio plot of carbohydrate absorbance peaks: **(A)** 1036 cm^– 1^; **(B)** 1050 cm^– 1^; **(C)** 1086 cm^– 1^; and **(D)** 1160 cm^– 1^ normalized to the lipid peak at 1740 cm^– 1^ taken from FT-IR data, symbols are the means from FT-IR spectra and error bars denote one standard deviation from the mean value.

According to the PC1 loadings plot (Figure 3) the main vibrational regions that contribute toward the separation of the samples included: 1655 cm^–1^ (amide I, C = O of proteins and peptides) (Maquelin et al., 2002); 1545 cm^–1^ (amide II, combination of in-plane N-H bending (60%) and C-N stretching (40%) of proteins, secondary structure of protein) (Lu et al., 2011); 1153 cm^–1^ (stretching vibrations of hydrogen bonded C-O groups; carbohydrates) (Pop et al., 2013; Simonova and Karamancheva, 2013); 1080 cm^–1^ (carbonyl groups in cell wall, glycopeptides); P = O stretching, P-O-C (P-O-P) of phospholipids and esters) (Filip et al., 2008); 1024 cm^–1^ (C-O bending and stretching typical of glycogen) (Lewis et al., 2010). The FT-IR spectra confirmed the PCA findings, with clear variance in the baseline corrected spectra apparent, which became more pronounced in the irradiated samples taken at the later time points (Supplementary Figure 4). Over the course of the experiment the irradiated samples showed increased spectral intensities from 1200 to 900 cm^–1^, which is indicative of an increase in total polysaccharides (Naumann, 2000; Ellis et al., 2012). Conversely, reduced spectral intensities were apparent in the amide I and II regions at 1655 cm^–1^ and 1545 cm^–1^ which indicates that there was a reduction in the total peptide content as the irradiated cultures age (Naumann, 2000; Ellis et al., 2012). The total carbohydrate band heights at 1160, 1086, 1050, and 1036 cm^–1^ were quantified and normalized by expressing them as a “ratio value” to the lipid band at 1740 cm^–1^, as there was no significant differences observed in this lipid band region. At day 4, the ratio value at 1160 cm^–1^ (Figure 4) for the irradiated sample was 1.35 (SD 0.11) compared to 1.43 (SD 0.08) in the equivalent control sample, showing that there was no significant difference in the polysaccharide levels during the irradiation treatment. The ratio value of the control samples did not vary much over the course of the sampling period, starting at 1.89 at day 4 with a slight reduction to 1.87 at day 16. The irradiated samples showed continued increases in the ratio value reaching 2.49 at day 16, a 1.85 fold increase compared to day 4. At day 16 there was a 1.97 fold increase in the polysaccharide signature of the irradiated samples compared the control. The carbohydrate bands at 1086, 1050, and 1036 cm^–1^ all showed the same trend in ratio values. The largest fold change between the day 16 samples was observed at the 1036 cm^–1^ band, which had a 2.69 fold increase in the irradiated samples.

### Total Carbohydrate Concentrations

**FIGURE 5:**
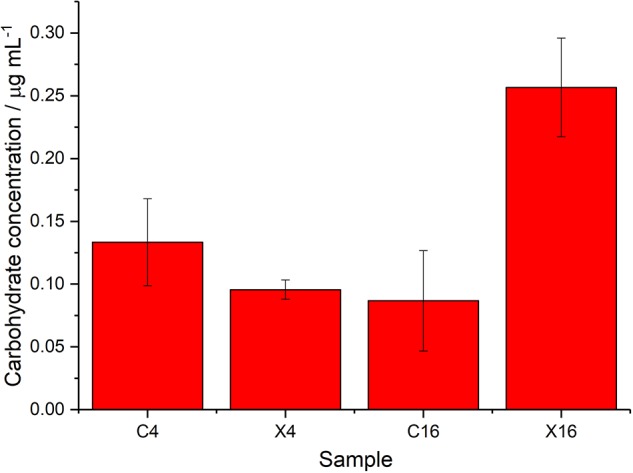
Total cell carbohydrate concentrations measured using Sigma-Aldrich kit, samples include controls (C), and irradiated (X) at day 4 and 16, bars are the mean values from 3 measurements and error bars denote standard deviations.

To investigate the FT-IR spectroscopy findings further, the total carbohydrate concentrations in the day 4 and day 16 samples (OD_600__nm_ normalized to 15) were determined (Figure 5). At day 4, the concentrations were 0.13 and 0.10 μg mL^–1^ for the control and irradiated samples, respectively. By day 16 the control sample had shown a slight reduction in carbohydrate levels to 0.09 μg mL^–1^, which is in agreement with the ratio plots taken from the FT-IR spectroscopy data. The irradiated samples showed an increase to 0.26 μg mL^–1^ at day 16 (2.69 fold increase), which is also in agreement with the FT-IR ratio plots. A comparison of the carbohydrate concentrations at day 16 showed a 2.96 fold increase in the concentration of the irradiated samples compared to the control.

### Fluorescent Light Microscopy Determination of Cell Morphology and Polysaccharide Staining

**FIGURE 6:**
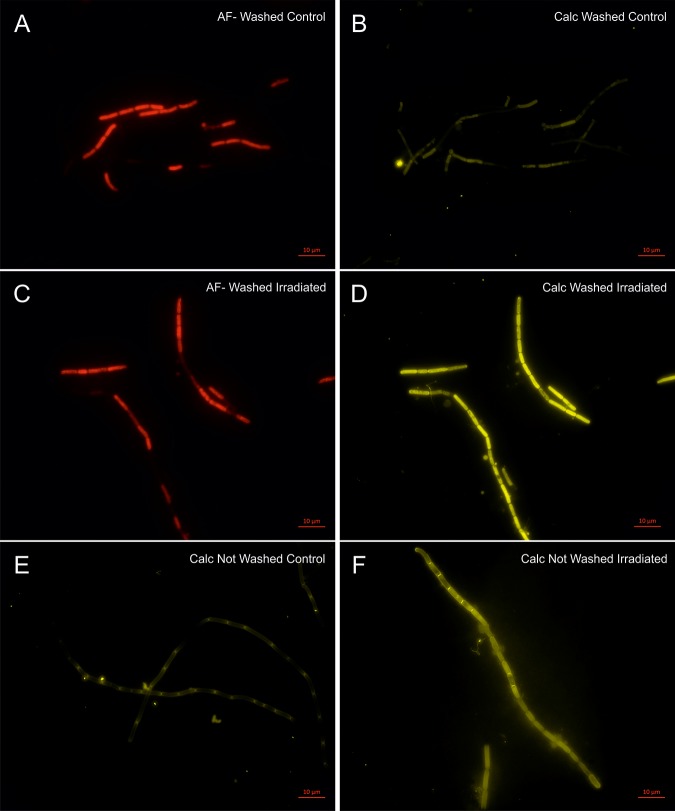
Light microscopy of *P. catenata* filaments at day 16: **A–D** were washed twice with normal saline [0.9 g L^– 1^ NaCl] **(A)** auto-fluorescence of control culture; **(B)** calcofluor white stained control culture; **(C)** auto-fluorescence of irradiated culture; **(D)** calcofluor white stained irradiated culture; **(E)** calcofluor white stained unwashed control culture; and **(F)** calcofluor white stained unwashed irradiated culture. Scale bar denotes 10 μm.

Calcofluor white stain was used to label β-linked polysaccharides associated with cells in the culture, to determine if the changes seen in the FT-IR spectra and carbohydrate analyses were due to upregulation of polysaccharides associated with cells of *P. catenata*. The auto-fluorescence of *P. catenata* was also noted throughout the experiment, which gave a qualitative assessment of the levels of photosynthetic pigments in the cells/filaments. There was little difference in both the auto-fluorescence and the binding of the calcofluor white stain to the *P. catenata* filaments in either the control or the irradiated cultures whilst they were still receiving the treatment at day 4 (Supplementary Figures 5A,C). However, by day 16 the auto-fluorescence seen across all of the *P. catenata* filaments in both treatments was more variable, with some cells lacking fluorescence altogether (Figures 6A,C). Interestingly, the cells that had been exposed to the irradiation treatment showed a greater degree of variability in the auto-fluorescence levels, with a higher proportion of the irradiated cells showing reduced fluorescence compared to the non-treated filaments. At day 4 the level of fluorescence with the calcofluor polysaccharide stain was comparable between the treated and non-treated cultures (Supplementary Figures 5B,D). The non-irradiated controls showed the same level of fluorescence with the calcofluor white stain at day 4 and day 16, suggesting similar levels of β-polysaccharides with time. The irradiated samples, however, showed increased levels of fluorescence of the calcofluor white stain at day 16 compared to the control cultures, providing evidence that irradiated *P. catenata* had higher levels of β-polysaccharides associated with the cell walls or extracellular mucilage (Figures 6B,D). Unwashed samples were also inspected using the calcofluor white stain, and the non-irradiated cells showed low levels of binding and fluorescence (Figure 6E). The stain was concentrated at the poles of the unwashed non-irradiated cells where they were connected within the filament. The unwashed irradiated cells showed the same elevated levels of fluorescence with the calcofluor stain as the washed samples. The calcofluor stain was also bound to extracellular material apparently associated with the unwashed irradiated *P. catenata* filaments, localized at the points where the cells in the filaments were connected (Figure 6F). This suggests that the cells were releasing β-polysaccharide containing materials into the supernatant which were removed upon washing.

## Discussion

The FGMSP located on the Sellafield site is colonized by microorganisms with diverse metabolic capabilities, including the potential to drive primary colonization by photosynthesis. During a microbial bloom in August 2016, a cyanobacterium belonging to the genus *Pseudanabaena* dominated the pond community (Foster et al., 2020). Here, we investigated the effect of X-ray irradiation on the growth and metabolism of a non-axenic culture of *P. catenata.* 16S rRNA gene sequencing revealed the presence of five OTUs within the *P. catenata* culture, which were consistent with genera identified in the FGMSP, making this culture an excellent representative of the pond community for model studies. The levels of radiation associated with the legacy SNFP are significant; Jackson et al. (2014) reported doses of 5.65 Gy h^–1^ associated with sludge and 0.15 mGy h^–1^ with the pond water. The dose will also be dynamic as the ponds are consistently purged with water and in pond handling activities change the radiation flux that the microorganisms are likely to be in contact with. This study assesses the effect of radiation on a *P. catenata* culture, which is related to the major photosynthetic microorganism found in a high pH and significantly radioactive legacy SNFP; here delivered at representative doses, over consecutive days.

Collectively the results of our experiments show that while the irradiation treatment was being administered to the culture there were no visible phenotypic differences observed compared to the control cultures. This suggests that the entire irradiated culture, including all microorganisms present, was able to fully tolerate the radiation dose administered. Differences between the two treatments only became apparent during the post-irradiation recovery period, and became more pronounced over time. The estimation of total biomass by the OD_600__nm_ and cell counts were the only measurements that remained comparable between the two treatments. Although the cell counts of *P. catenata* increased over time, the recorded Chl-a concentrations did not increase in line with cell numbers. Inspection of the auto-fluorescence at day 16, when the differences were greatest, showed varied levels of fluorescence across filaments. This suggests that within a filament of *P. catenata*, cells were showing different levels of photosynthetic capacity. The differences between the Chl-a concentrations, the cell numbers, and optical density readings suggest that irradiated *P. catenata* filaments had fewer photosynthetically active cells than in the control cultures. A study by Sigee et al. (2007) highlighted that estimations of total abundance of cyanobacterial populations might be misleading as some organisms are at different stages of growth and may be in a senescent state. Thus, the increase in cell numbers predicted by the optical density measurements alone may not match the number of viable and actively dividing cells.

Previous studies investigating the effect of ionizing radiation on axenic cultures of cyanobacteria have reported similar drops in chlorophyll concentrations, but after much higher doses from a ^60^Co-gamma radiation source. El-Fatah Abomohra et al. (2016) reported up to a 25% reduction in the chlorophyll concentrations of *Arthrospira platensis* 15 days after exposure to 2.5 kGy of radiation. This coincided with a reduction in total biomass production by 34%. At lower doses of 1 and 1.5 kGy, no recorded drop in biomass was reported, however, chlorophyll concentrations were reduced by 8 and 12%, respectively. The effects of irradiation treatments on chlorophyll production is varied, however, as Badri et al. (2015) reported no significant impacts on chlorophyll when exposing *Arthrospira* cultures to similar doses used by El-Fatah Abomohra et al. (2016). The authors reported a reduction in the antenna pigments allophycocynanin and phycocynanin in addition to an increasing lag phase in growth as the dose of radiation increased. *Anabaena* cultures exposed to gamma irradiation showed bleaching of their pigments immediately after exposure to 6 kGy, with a 42.5% reduction in chlorophyll-a concentration. However, all cultures were able to recover following irradiation, although longer lag phases were observed at higher doses (Singh et al., 2013). Several studies have reported that low doses of ionizing radiation can stimulate the growth of cyanobacteria, for example Wang et al. (1998) demonstrated this with an *Arthrospira* spp. at 500 Gy, whilst several studies report the enhanced growth of a *Synechococcus* spp. at dose rates of 20 mGy y^–1^ (Conter et al., 1984, 1986). The stimulatory effect of lower chronic doses of ionizing radiation could offer a plausible explanation for the continued increase in cell numbers we observed despite the drop in chlorophyll concentration and auto-fluorescence in *P. catenata*. These studies show that the effect of radiation can be varied and that photosynthetic pigments are affected but the dose at which this is observed differs between species. The reduction in the concentration of Chl-a, alongside other processes to downregulate photosynthetic activity, could be an adaptive response to environmental stress in order to prevent photodamage and the accumulation of reactive oxygen species, that can otherwise form through uncontrolled photosynthetic electron flow (Latifi et al., 2009). Regardless, the doses applied in the current work (total of 95 Gy) were selected to be broadly representative of likely doses within the FGMSP and thus tailored to provide relevant insights. Further experimental work utilizing transcriptomic or proteomic techniques could help determine the ability of this cyanobacterium to continue to proliferate despite the reduction in Chl-a, this is, however, out of the scope of the current study.

The collected FT-IR spectral data, ratio plots of vibrational features, total cell carbohydrate concentrations and calcofluor white staining all show an overall increase in carbohydrate production over time in the irradiated cultures. From the FT-IR spectra it is not possible to determine which organisms are responsible for the differences observed, as the interrogation beam has a diameter of ∼1 mm and so measures the whole microbial community. The wavenumbers observed in the PCA loadings plots, contributing to the shifts seen in the PCA scores plots, indicate that there are potentially changes associated with intracellular and extracellular polysaccharides. The wavenumber 1024 cm^–1^ is indicative of glycogen which is a common storage molecule in cyanobacteria and some bacteria. Nutrient stress has been shown to result in increased storage of glycogen in *Synechococcus* species, however, this coincides with a reduction of growth (Klotz et al., 2016), which is not observed in the current study. Calcofluor white stain is commonly used to identify the presence of chitin, a β-polysaccharide found in fungal cell walls, but it is also used to stain a variety of β-polysaccharides (Anderson et al., 2010; Dunker et al., 2017). In our study, the calcofluor white stain was associated with the outer surface of the *P. catenata* cells, suggesting that there is an increase in β-polysaccharides associated with extracellular polymeric substances (Singh et al., 2013). It should be noted that doses of 0.5 – 1.5 kGy gamma irradiation have been shown to result in the increased production of carbohydrates in *Arthrospira* spp. in other studies (El-Fatah Abomohra et al., 2016). It is thought that the polymeric substances provide an array of functions including increasing cell buoyancy, binding metals, accumulating nutrients, aggregation of cells to one another, the formation of biofilms on surfaces and a barrier to protect against environmental stress (Nobles et al., 2001; Xu et al., 2013; Gao et al., 2015). It is not known whether the microorganisms in the legacy SNFP produce such polymeric substances; however, the similarities between the community profile in the pond and the culture used in this study suggest that this is feasible and warrants further investigation. The increased production of polysaccharides/polymeric substances by organisms in the pond could provide a mechanism to protect microorganisms from the damaging effects of reactive oxygen species, which are formed as a result of the radiolysis of water (Kottemann et al., 2005; Gao et al., 2015).

The presence of polysaccharides or polymeric substances associated with the microorganisms would also have implications for the fate of radionuclides in the pond and downstream processes. Cationic metals are able to adsorb to negatively charged functional groups on the surfaces of the microorganisms and polysaccharide containing mucilage of some cyanobacteria at neutral to alkaline pH (Gadd, 1990, 2009; Javanbakht et al., 2014; Decho and Gutierrez, 2017). Extracellular polymeric substances also have the ability to trap organic and inorganic colloids and nanoparticles, which are thought to be present in the pond (Maher et al., 2016; Decho and Gutierrez, 2017; Neil et al., 2018). The same experimental set-up described in this study was used recently to investigate the interaction of ^90^Sr with the cell free medium from irradiated and control cultures (Ashworth et al., 2018). All of the ^90^Sr remained in solution when it was added to the cell free medium from the control cultures, whilst the irradiated samples resulted in the removal of approximately 10% of ^90^Sr from solution. Analyses of the supernatants showed higher total carbon levels in the control cultures (324 mg L^–1^) compared to the medium from the irradiated cultures (162 mg L^–1^). The lower levels of TOC in the irradiated medium observed was surprising, particularly as the calcofluor staining presented in this study indicates the presence of extracellular material in unwashed samples which are not present following centrifugation and washing. The reduced TOC in the medium from the irradiated cultures suggests that irradiated medium either has modified functional groups which better facilitate interactions with ^90^Sr or that the irradiation treatment has resulted in the secretion of additional metabolites not present in the control samples. As noted by Ashworth et al. (2018) the level of interaction although being low is worth exploring further, as is the interaction of strontium in the presence of the microorganisms in the culture.

This study provides an insight into broad scale changes in the metabolism of a microbial community dominated by *P. catenata* in response to doses of irradiation. The metabolic responses revealed by FT-IR spectroscopy are representative of a culture-wide response, and it is therefore difficult to attribute to an individual organism. However, *P. catenata* specific responses were observed with the decline in photosynthetic pigments, whilst the calcofluor staining showed some of the changes observed in the polysaccharide levels are most likely attributed to this organism. Furthermore, the data presented here show that FT-IR spectroscopy would be a very powerful tool to investigate broad scale changes in the metabolic state of the pond community *in situ*. The utilization of additional metabolomics techniques, such as GC-MS on cell extracts and spent medium would provide further more detailed information about the different metabolic pathways, and metabolite levels that support such irradiation tolerance. In addition, other molecular techniques such as metatranscriptomics and metaproteomics, would provide information about changes in gene expression and thus provide insights into the reduced amide spectral intensities seen in the FT-IR spectroscopy data.

The FGMSP on the Sellafield site is currently being decommissioned, which involves amongst other things the removal of waste stored in the pond. In order to safely and efficiently carry out routine pond operations, visibility within the pond must be maintained. The presence of microorganisms in the pond has the potential to reduce visibility and cause delays in the on-site operations, particularly during microbial bloom events. Whilst the microbial community has recently been determined (Foster et al., 2020), little was known about the survival mechanisms the organisms used to colonize the pond. The results presented in this study provide new insights into the adaptive response of a *P. catenata* dominated culture to FGMSP relevant doses of irradiation. As noted above, the identification of increased cell polysaccharide levels is of importance since elevated polysaccharide levels could affect the behavior and fate of key radionuclides present in the pond (Gadd, 1990). High levels of polysaccharide containing material could also play a role in supporting the growth of the heterotrophic microbial community whilst providing the microorganisms a protective barrier against the environment (Song et al., 2016). Analysis of microbial communities inhabiting SNFPs so far, indicate that the communities are specific to individual ponds. Recently the dominant algal species causing microbial blooms in a near neutral pH SNFP on the Sellafield Ltd., site, was shown to synthesize large quantities of the carotenoid astaxanthin, which is known to have antioxidant properties (MeGraw et al., 2018). The research carried out in this current study and in McGraw et al. (MeGraw et al., 2018) indicate that the adaptive response of the microbial communities is unique to the specific microorganism and the SNFP that they have colonized. A greater understanding of the microbial responses to the radiation and other stresses they encounter in the legacy pond will help to optimize control strategies used on site to control the microbial load in the pond and prevent blooms occurring during the planned decommissioning of the FGMSP over the next 20 + years. This study also provides further information about the response of microorganisms to doses of ionizing radiation that have not previously been studied, but which are relevant to critical engineered environments, including a wider range of nuclear facilities worldwide. In this context, understanding how microorganisms able to tolerate high radiation doses interact with key radionuclides, could also be key to developing innovative biotechnological approaches for treating pond waters and nuclear effluents, and is therefore an area of intense interest worldwide.

## Data Availability Statement

The raw data obtained in this research were deposited to NCBI SRA (Sequence Read Archive; http://www.ncbi.nlm.nih.gov/sra/) under the project accession number: PRJNA607014.

## Author Contributions

LF was the principal author, carried out the experimental work and data analysis. HM collected and analyzed the FT-IR data. CB carried out the DNA sequencing and reviewed the manuscript. DS, JP, RG, and KM developed the concept and reviewed the manuscript. JL developed the concept and extensively reviewed the manuscript.

## Conflict of Interest

The authors declare that the research was conducted in the absence of any commercial or financial relationships that could be construed as a potential conflict of interest.
