# Supplementary material for: Radiation Tolerance of Pseudanabaena catenata, a Cyanobacterium Relevant to the First Generation Magnox Storage Pond
Source: Front Microbiol. 2020 Apr 7;11:515. doi: 10.3389/fmicb.2020.00515 (PMC7154117; doi:10.3389/fmicb.2020.00515)
Supplement: Supplementary file 1 [file Data_Sheet_1.docx]

## Supplementary material

## Microbial community analysis of the *P. catenata* culture determined by 16S rRNA gene sequencing


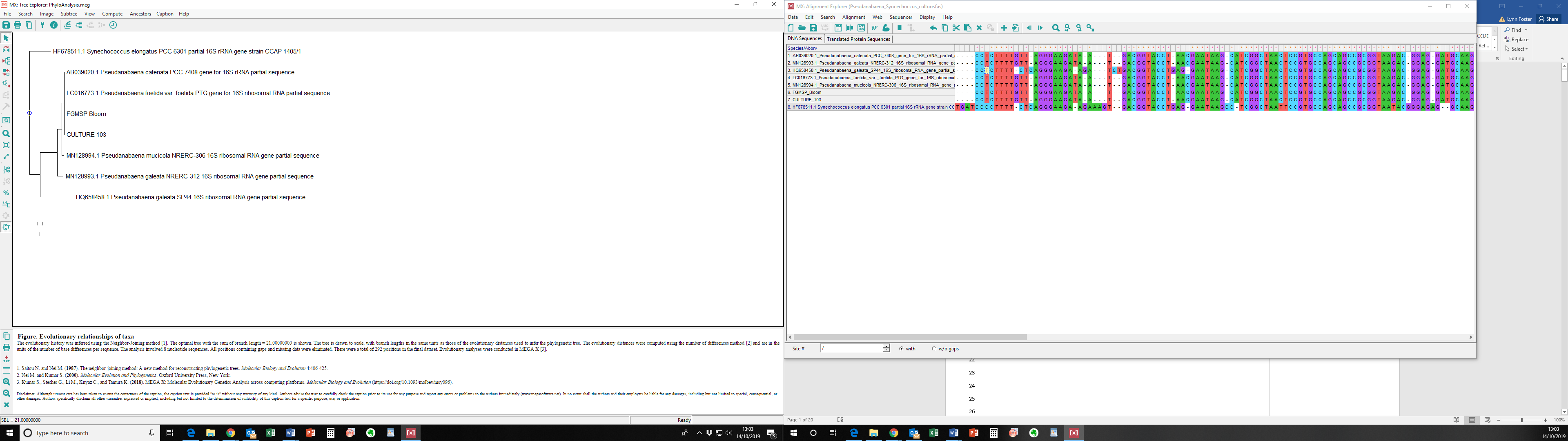


**Supplementary Figure 1: Evolutionary relationships of taxa. The evolutionary history was inferred using the Neighbor-Joining method [1]. The optimal tree with the sum of branch length = 0.17987446 is shown. The percentage of replicate trees in which the associated taxa clustered together in the bootstrap test (500 replicates) are shown next to the branches [2]. The tree is drawn to scale, with branch lengths in the same units as those of the evolutionary distances used to infer the phylogenetic tree. The evolutionary distances were computed using the Kimura 2-parameter method [3] and are in the units of the number of base substitutions per site. The rate variation among sites was modeled with a gamma distribution (shape parameter = 0.05). The analysis involved 8 nucleotide sequences. All positions with less than 95 % site coverage were eliminated. That is, fewer than 5% alignment gaps, missing data, and ambiguous bases were allowed at any position. There were a total of 292 positions in the final dataset. Evolutionary analyses were conducted in MEGA X [4].**

1. Saitou N. and Nei M. (**1987**). The neighbor-joining method: A new method for reconstructing phylogenetic trees. *Molecular Biology and Evolution* **4**:406-425.

2. Felsenstein J. (**1985**). Confidence limits on phylogenies: An approach using the bootstrap. *Evolution* **39**:783-791.

3. Kimura M. (**1980**). A simple method for estimating evolutionary rate of base substitutions through comparative studies of nucleotide sequences. *Journal of Molecular Evolution* **16**:111-120.

4. Kumar S., Stecher G., Li M., Knyaz C., and Tamura K. (**2018**). MEGA X: Molecular Evolutionary Genetics Analysis across computing platforms. *Molecular Biology and Evolution* (https://doi.org/10.1093/molbev/msy096).

Since the *P. catenata* culture was not axenic, 16S rRNA gene sequencing was carried out on the cultures to determine the community diversity of the culture. A comparison of the prokaryotic community at day 16 between the irradiated and control samples was made to determine what microorganisms were present and to see if the irradiation treatment resulted in shifts in the diversity of the culture with and without irradiation. The culture consisted of 9 OTUs including *P. catenata* between the two sets of culture. This showed that the irradiation treatment did not result in a significant shift in phylogenetic diversity (Supplementary Fig 1). As expected the most abundant OTU in both cultures was affiliated with a *Pseudanabaena* species*,* which comprised 30.7 % (control) and 32.7 % (irradiated) of the total community. The remainder of the OTUs were associated with the phyla *Bacteriodetes* (37.3 % control; 36.5 % irradiated; 2 OTUs) and *Proteobacteria* (32.0 % control; 30.7 % irradiated; 6 OTUs). A comparison of the microorganisms identified in the *P. catenata* culture showed strong similarities to those identified in legacy SNFP samples, particularly during the bloom. Of the 9 OTUs identified in the culture, 5 were affiliated with genera identified in the main pond namely: species of *Pseudanabaena*; *Flavobacterium*; *Porphyrobacter*; *Rhodobacter*; and *Hydrogenophaga*^26^. In addition, species of *Sediminibacterium* and *Lacibacterium* were observed in samples from an auxiliary pond^26^, which feeds into the high pH legacy SNFP on the Sellafield Ltd site, making this culture highly representative of the SNFP.


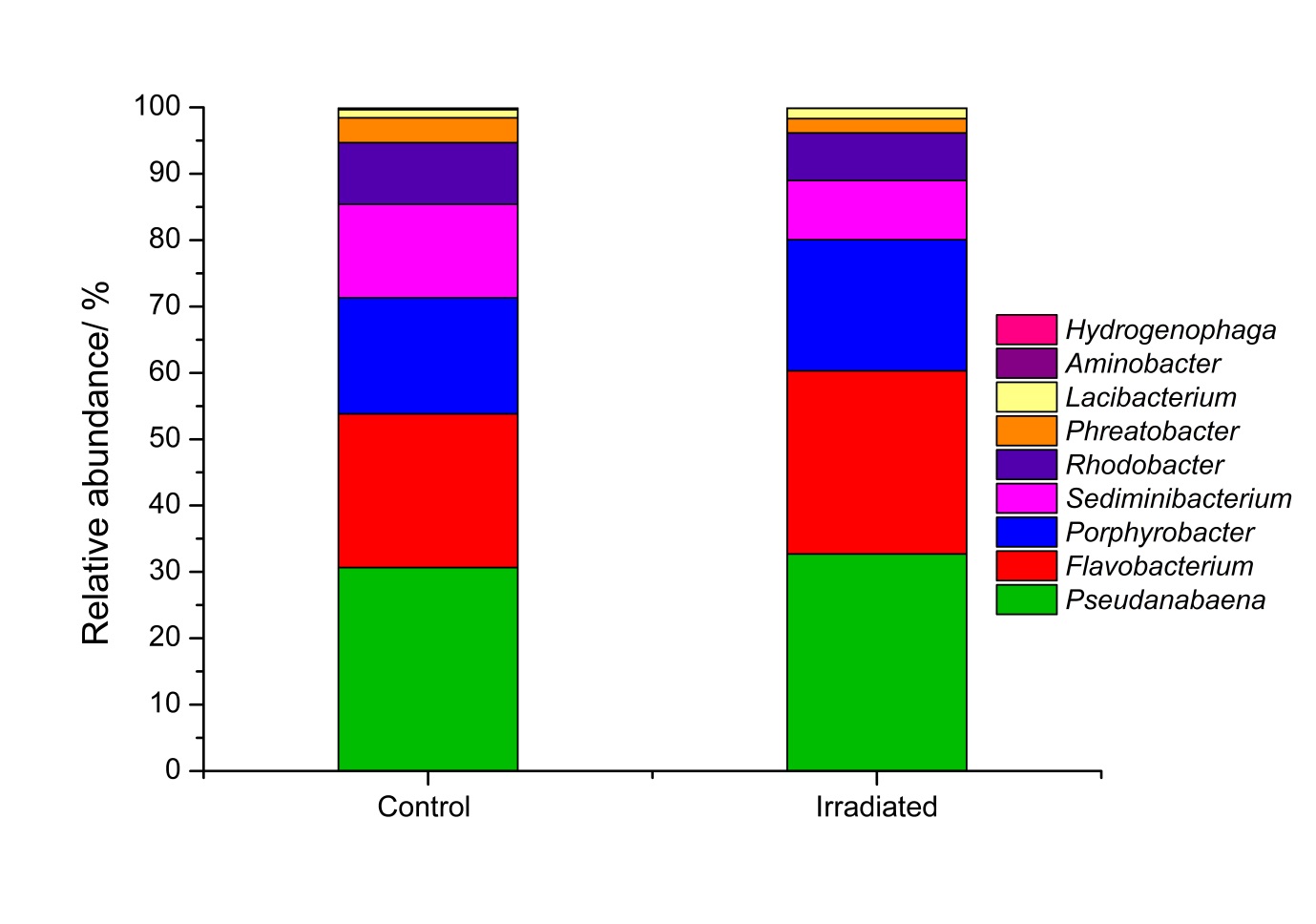


Supplementary Figure 2: Genus-level microbial community analysis of the *P. catenata* culture at day 16, comparing the 16S rRNA gene irradiated community profile to that of the untreated control.


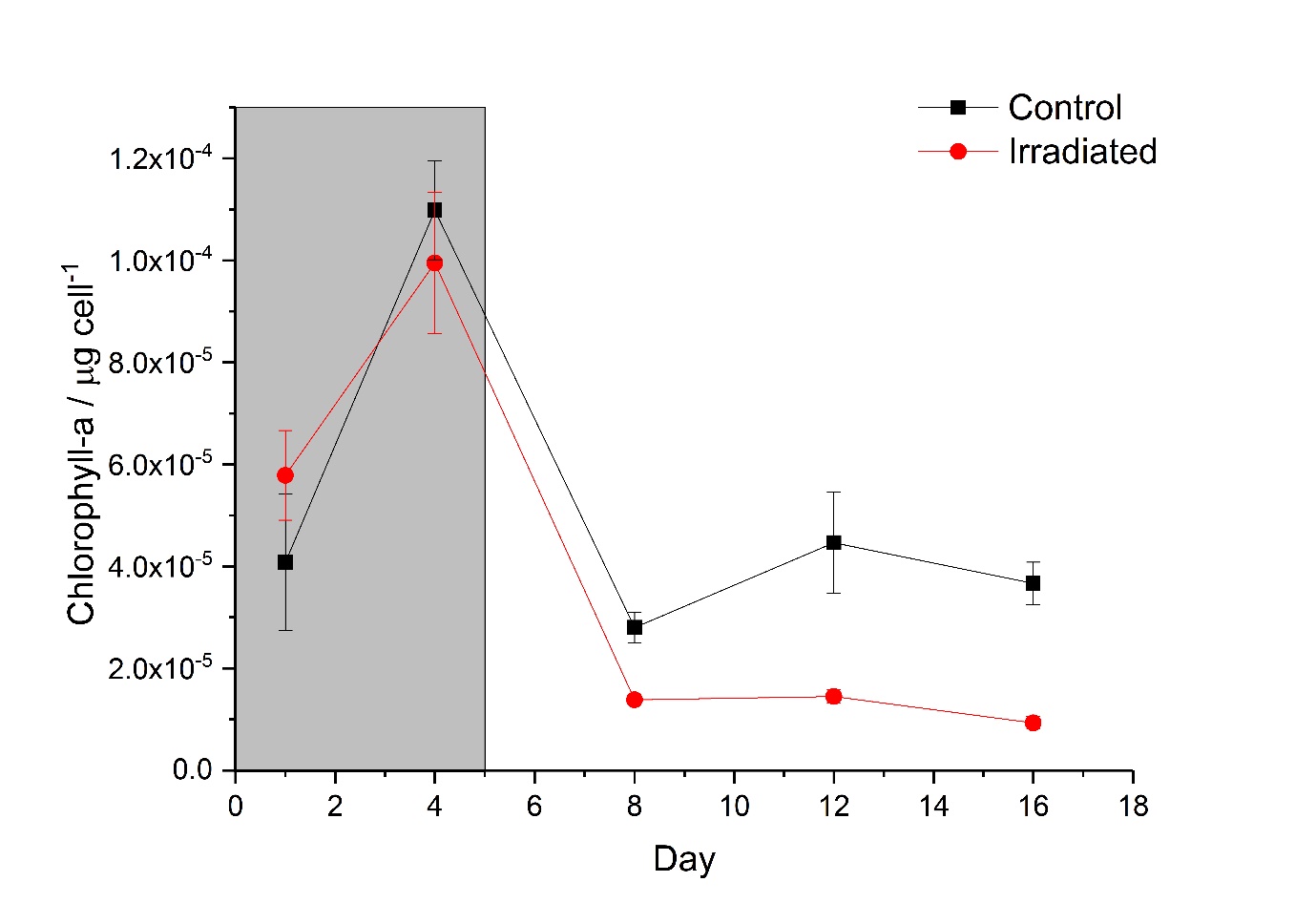


Supplementary Figure 3: Chlorophyll-*a* concentration (μg L^-1^) normalised to average cell number. The grey box indicated the period where irradiation treatment was being administered. Error bars denote standard deviations of three replicates


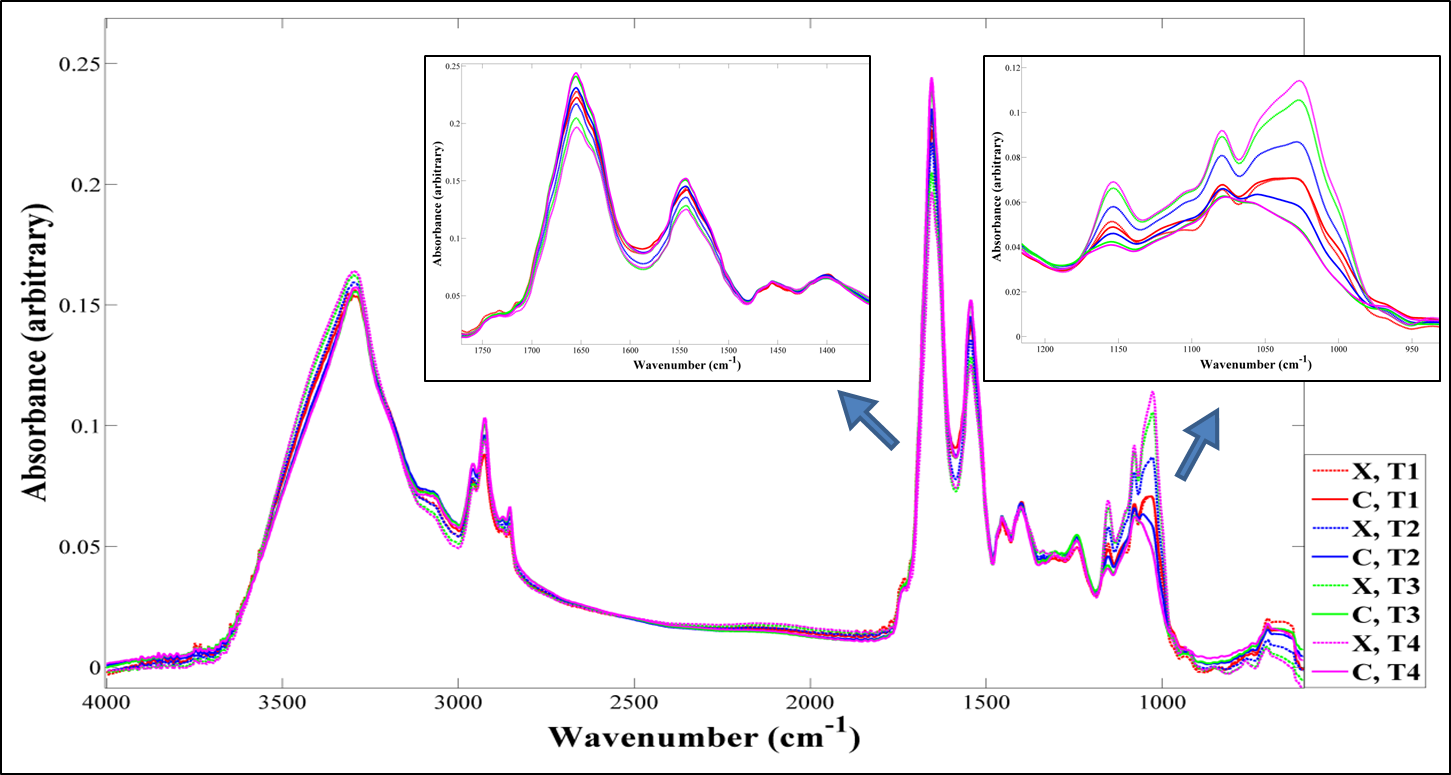


Supplementary Figure 4: Average absorbance FT-IR spectra, with zooms of important spectral features. X denotes irradiated samples and C denotes control samples. T1= day 4; T2= day 8; T3= day 12; and T4= day 16


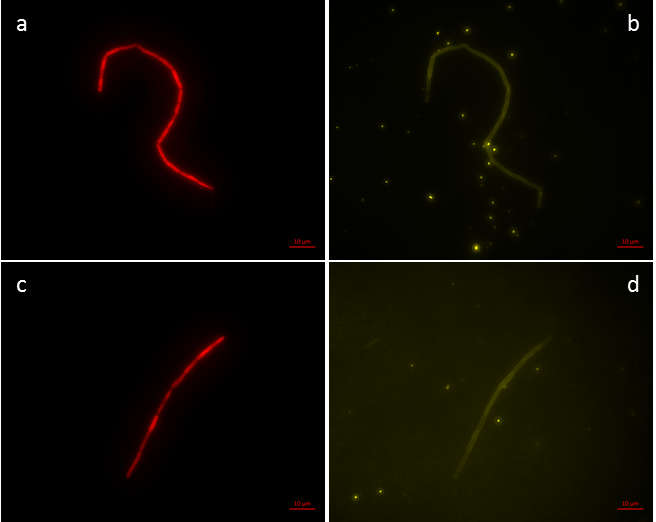


Supplementary Figure 5: Light microscopy of *P. catenata* filaments at day 4 after washing in normal saline: a) autofluorescence of control culture; b) calcofluor white stained control culture; c) autofluorescence of irradiated culture; and d) calcofluor white stained irradiated culture. The scale bars = 10 μm
